# Mechanical Thrombectomy Versus Anticoagulation in Intermediate-Risk Pulmonary Embolism: A Systematic Review and Meta-Analysis

**DOI:** 10.1007/s00270-026-04423-5

**Published:** 2026-03-22

**Authors:** James M. Chan, Valeria V. Varela Betancourt, Julia Mosqueira Almeida, Anggie L. Renteria Chamorro, Li H. Tan, Elisa Licari, Hui Yin Lim, Goran Mitreski, Hong Kuan Kok

**Affiliations:** 1 Faculty of Medicine, Dentistry and Health Sciences, University of Melbourne, Melbourne, Australia; 2 Department of Intensive Care and Hyperbaric Medicine, The Alfred Hospital, Melbourne, VIC Australia; 3 School of Medicine, National University of Colombia, Bogotá, Colombia; 4 Faculdade de Medicina de Barbacena, FAME/FUNJOB, Barbacena, Brazil; 5 Universidad Santiago de Cali, Palmira, Colombia; 6 Northern Pathology Victoria, Northern Health, Melbourne, Australia; 7 Department of Haematology, Northern Health, Melbourne, Australia; 8 Northern Clinical Diagnostics and Thrombovascular Research (NECTAR) Group, Northern Health, Melbourne, Australia; 9 School of Health and Biomedical Sciences, RMIT University, Melbourne, Australia; 10 Interventional Radiology Service, Northern Imaging Victoria, Northern Health, Melbourne, Australia

**Keywords:** Pulmonary embolism, Mechanical thrombectomy, Anticoagulation, Clinical outcomes

## Abstract

**Purpose:**

We performed a systematic review and meta-analysis comparing mechanical thrombectomy to anticoagulation in patients with intermediate-risk pulmonary embolism (PE) focusing on patient-centred outcomes.

**Materials and methods:**

PubMed, Embase and Cochrane databases were searched from inception to November 2025 for randomised controlled trials and observational studies comparing mechanical thrombectomy (with or without anticoagulation) to anticoagulation alone in patients with intermediate-risk PE. The main outcomes were all-cause in-hospital mortality, all-cause 30-day mortality, hospital length of stay and ICU length of stay.

**Results:**

We identified seven studies, comprising 2699 patients from one randomised controlled trial and six observational studies. Mechanical thrombectomy, compared to anticoagulation, was associated with significantly lower incidence of all-cause 30-day mortality (OR 0.09; 95% CI 0.02–0.41; *p* = 0.002; *I*^2^ = 0%). There was no difference in all-cause in-hospital mortality (OR 0.62; 95% CI 0.19–2.03; *p* = 0.29; *I*^2^ = 23%), hospital length of stay (mean difference − 1.85 days; 95% CI − 4.60 to 0.89 days; *p* = 0.13; *I*^2^ = 91%) or ICU length of stay (mean difference − 0.48 days; 95% CI − 2.62 to 1.67 days; *p* = 0.53; *I*^2^ = 84%).

**Conclusion:**

In patients with intermediate-risk PE, mechanical thrombectomy was associated with a lower incidence of all-cause 30-day mortality compared to anticoagulation. However, as the majority of the included studies were observational, these findings should be interpreted with caution and warrant confirmation with further high-quality randomised controlled trials.

**Graphical Abstract:**

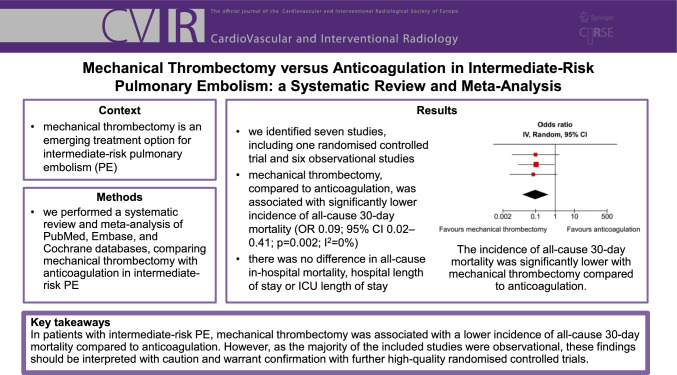

**Supplementary Information:**

The online version contains supplementary material available at 10.1007/s00270-026-04423-5.

## Introduction

Intermediate-risk pulmonary embolism (PE) refers to PE with right ventricular dysfunction or elevated cardiac troponin levels but without haemodynamic instability [1] and is associated with an early mortality rate of around 1–4% [1, 2]. The mainstay treatment for acute intermediate-risk PE is systemic anticoagulation [1, 3]. Systemic thrombolysis is reserved for patients with high-risk PE and is not routinely used in intermediate-risk PE due to the risk of major bleeding and the absence of a clear mortality benefit [3, 4]. Consequently, catheter-based reperfusion strategies have emerged as alternative management options for intermediate-risk PE [5], particularly in patients in the intermediate–high-risk category with clinical features concerning for deterioration. These strategies most commonly include catheter-directed thrombolysis, which involves the endovascular placement of a catheter within the pulmonary vasculature to deliver thrombolytic agents directly into the thrombus, and mechanical thrombectomy, which uses catheter-based devices to physically retrieve and/or aspirate the clot to restore pulmonary arterial flow [5–7].

A limited number of randomised controlled trials show a potential benefit of mechanical thrombectomy over anticoagulation alone [8] and other endovascular techniques [7] in intermediate-risk PE. Catheter-based techniques appear to rapidly improve right ventricle dysfunction [8, 9]. However, their effect on short-term mortality remains less clear. While some studies suggest low rates of short-term mortality [10], findings across the literature are inconsistent [8, 11–13]. The 2019 European Society of Cardiology (ESC)/European Respiratory Society (ERS) guidelines suggest catheter-based treatments should be considered in intermediate-risk PE patients only when there is haemodynamic deterioration despite anticoagulation [1]. These recommendations are based on low-level evidence [1] and were developed prior to the availability of contemporary thrombectomy techniques [13] and data available from recent registries and randomised studies [6, 8]. While further randomised trials are currently underway [6], to date, only a single randomised controlled trial has directly compared mechanical thrombectomy to anticoagulation in the intermediate-risk PE population [8].

Several meta-analyses published in the last few years have compared mechanical thrombectomy and anticoagulation in acute PE [14–16]. However, these analyses have frequently combined PE populations across multiple risk groups (such as high-risk and intermediate-risk PE) [15–17], limiting their applicability to intermediate-risk PE patients specifically. In addition, some were conducted prior to the publication of the most recent randomised controlled trial and large registry data directly comparing mechanical thrombectomy with anticoagulation [8, 11–13, 17, 18]. In light of the ongoing uncertainty in this area and the new emerging studies, we performed an updated systematic review and meta-analysis on mechanical thrombectomy versus anticoagulation in patients with intermediate-risk PE. By synthesising the latest available studies focused on patient-centred outcomes, this analysis seeks to inform future updates of clinical practice guidelines by providing a more robust evidence base to identify subgroups most likely to derive clinical benefit from mechanical thrombectomy.

## Methods

### Eligibility Criteria and Outcomes

We included studies that met the following eligibility criteria: (1) randomised controlled trials or observational studies; (2) that compared mechanical thrombectomy (with or without anticoagulation) to anticoagulation; (3) in patients with intermediate-risk PE; and (4) reported at least one outcome of interest. The subgroups of intermediate-risk PE described in the 2019 ESC/ERS guidelines—intermediate–low and intermediate–high-risk PE—were included [1]. Additionally, studies involving submassive PE were included given its similarity to intermediate–high-risk PE [3]. Although intermediate–low risk, intermediate–high risk, and submassive PE are not entirely identical entities, they represent a spectrum of haemodynamically stable PE with evidence of right ventricular strain and/or myocardial injury. Given this, we considered pooling them appropriate to reflect real-world clinical decision-making. We excluded studies that did not directly compare mechanical thrombectomy with anticoagulation, studies that lacked a clearly defined intermediate-risk PE population, studies that did not report at least one outcome of interest, studies with fewer than ten patients per group, review articles, editorials, commentaries, case reports, case series, single-arm studies, letters, conference abstracts, studies not published in full, animal studies, paediatric studies and non-English language studies. Non-English studies were excluded due to resource constraints and lack of formal translation support, which precluded reliable data extraction and quality assessment. Outcomes included all-cause in-hospital mortality, all-cause 30-day mortality, hospital length of stay and ICU length of stay. We report all-cause in-hospital mortality; however, we acknowledge that discharge represents a competing risk, as patients leave the hospital either due to recovery or death. This limitation should be considered when interpreting the in-hospital mortality results.

### Search Strategy and Data Extraction

This systematic review and meta-analysis was performed and reported in accordance with the Cochrane Collaboration Handbook for Systematic Review of Interventions [19, 20] and the Preferred Reporting Items for Systematic Reviews and Meta-Analysis (PRISMA) Statement [20] guidelines. We systematically searched PubMed, Embase and the Cochrane Central Register of Controlled trials from inception to November 2025 for eligible studies. The full search strategies for each database are shown in Supplementary Material, section 1 Following removal of duplicates, title and abstracts were screened by two authors (JMC and JMA) independently and in a blinded manner. Subsequently, full texts were screened by two authors (JMC and VVVB) also independently and in a blinded manner. Screening was performed using the Rayyan software platform [21]. Data extraction was conducted independently by two authors (VVVB and ALRC), and any discrepancies were resolved after consultation with a third author (JMC). The prospective meta-analysis protocol was registered on PROSPERO on 19 November 2025 under protocol CRD420251184248.

### Quality Assessment

Risk of bias was assessed with the RoB-2 tool [22] for randomised controlled trials and the ROBINS-I version 2 [23] tool for observational studies. Publication bias was analysed by funnel plot analysis of the point estimates in relation to study weights.

### Statistical Analysis

Odds ratios (OR) with 95% confidence intervals were used to compare treatment effects for binary outcomes. For observational studies, adjusted odds ratios were preferentially extracted and pooled when reported, as these account for measured confounders. If adjusted estimates were not available, unadjusted odds ratios were used. For binary outcomes, studies with zero events in one treatment arm were included using a continuity correction of 0.5, as implemented in Review Manager. Studies with zero events in both treatment arms were excluded from the pooled odds ratio analysis, as effect estimates cannot be calculated in such cases and these studies do not contribute information regarding relative treatment effect. Continuous outcomes were compared using mean differences. For continuous variables where mean and standard deviation were not available, medians and IQRs were used to calculate the mean and standard deviation using the method outlined in the Cochrane Handbook for Systematic Reviews of Interventions [19, 24]. For pooled analyses using adjusted odds ratios, we employed an inverse-variance method with random effects (using restricted maximum likelihood). For continuous data, we used an inverse-variance approach with mean differences and random effects and also estimated via restricted maximum likelihood. We assessed heterogeneity with the *I*^2^ statistics. *I*^2^ values from 0 to 25%, 25–75% and 75–100% were considered low, moderate and high heterogeneity, respectively. We used the DerSimonian and Laird random effects model. We used Review Manager (Cochrane, The Cochrane Collaboration, Denmark) [25] for statistical analysis. For outcomes demonstrating high heterogeneity, leave-one-out sensitivity analyses were performed to assess the impact of individual studies and to evaluate the robustness of the overall pooled effect estimate.

## Results

### Study Selection and Baseline Characteristics

**Fig. 1:**
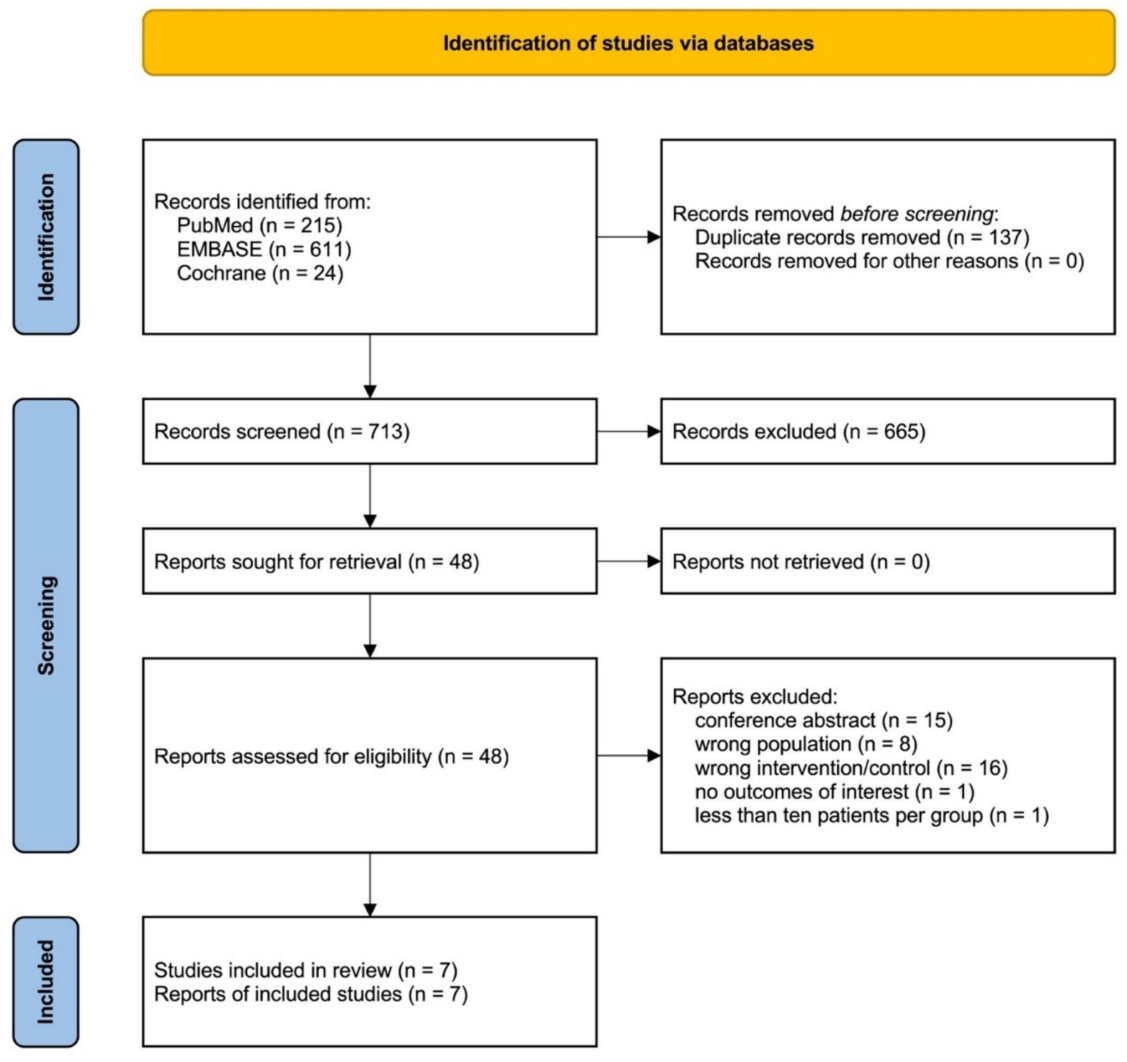
PRISMA flow diagram of study screening and selection

The initial search yielded 850 results. After removal of 137 duplicate records and 665 records based on title and abstract screening, 48 remained and were fully reviewed (Fig. 1). Of these, seven studies were included [8, 9, 11–13, 18, 26], comprising 2,699 patients from one randomised controlled trial and six observational studies. Three studies [8, 18, 26] exclusively included patients with intermediate–high risk or submassive PE, and four studies [9, 11–13] included patients with intermediate-risk PE more broadly. A total of 1362 (50.46%) patients underwent mechanical thrombectomy and 1337 (49.54%) patients received anticoagulation. In the mechanical thrombectomy group, various devices were used including the FlowTriever (Inari Medical, Irvine, CA), the Lightning Flash 16F catheter system (Penumbra, Alameda, CA) and the Indigo Aspiration System (Penumbra, Alameda, CA). In the anticoagulation group, a range of medications were used, including enoxaparin, dalteparin and unfractionated heparin. Follow-up time ranged from 7 days to 4.2 months. Study characteristics are described in Table 1.

**Table 1:** Baseline characteristics of included studies

Study	Design	Patients MT/AC	Population	MT treatment	AC treatment	Age (years) MT/AC	Female (n, %) MT/AC	Follow-up time
Lookstein [8]	RCT	47/53	Intermediate–high-risk PE	Lightning Flash 16F catheter system (Penumbra, Inc, Alameda, CA)	UFH or LMWH	59.5 (13.22)/61.2 (14.19)*	18, 38.3%/28, 52.8%	7 days
Khazi [26]	Observational (retrospective)	41/82	Submassive PE	Inari FlowTriever	NA	58.58 (14.18)/ 59.28 (16.24)*	25, 61%/ 35, 42.7%	30 days
Nolan [18]	Observational (retrospective)	41/200	Intermediate–high-risk PE (subgroup)	Inari FlowTriever or Indigo Aspiration System	UFH, LMWH or bivalirudin	NA/NA	NA/NA	30 days minimum
Patel [13]	Observational (retrospective)	1030/515	Intermediate-risk PE	CAVT or other MT	NA	CAVT: 62.8 (15.53)/Other MT: 63.1 (14.67)/AC: 65.7 (16.18)*	CAVT: 246, 47.8%/Other MT: 246, 47.8%/AC: 248, 48.2%	NA
Teo [11]	Observational (prospective)	94/176	Intermediate-risk PE	Inari FlowTriever	NA	60.48 (15.1)/62.3 (16.7)*	41, 43.6%/94, 53.4%	3 months
Wang [9]	Observational (retrospective)	28/119	Intermediate-risk PE	16 and 20 Fr catheters manufactured by Chenxing Medical Devices Co., Ltd. and Shanghai Rongmai Medical Technology Co., Ltd	enoxaparin or dalteparin	64 [55–70]/65 [59–70]^a^	18, 64.3%/62, 52.1%	4.2 [3.1, 5.4] months^a^
Zhang [12]	Observational (retrospective)	81/192	Intermediate-risk PE	Inari FlowTriever	NA	57.2 (13.4)/61.7 (17.7)*	39, 48.1%/109, 56.8%	30 days^a^

AC Anticoagulation, CAVT Computer-assisted vacuum thrombectomy, LMWH Low molecular weight heparin, MT Mechanical thrombectomy, NA Not available, PE Pulmonary embolism, RCT Randomised controlled trial, UFH Unfractionated heparin

^*^mean and standard deviation

^a^median and interquartile range

### Pooled Analyses of All Included Studies

**Fig. 2:**
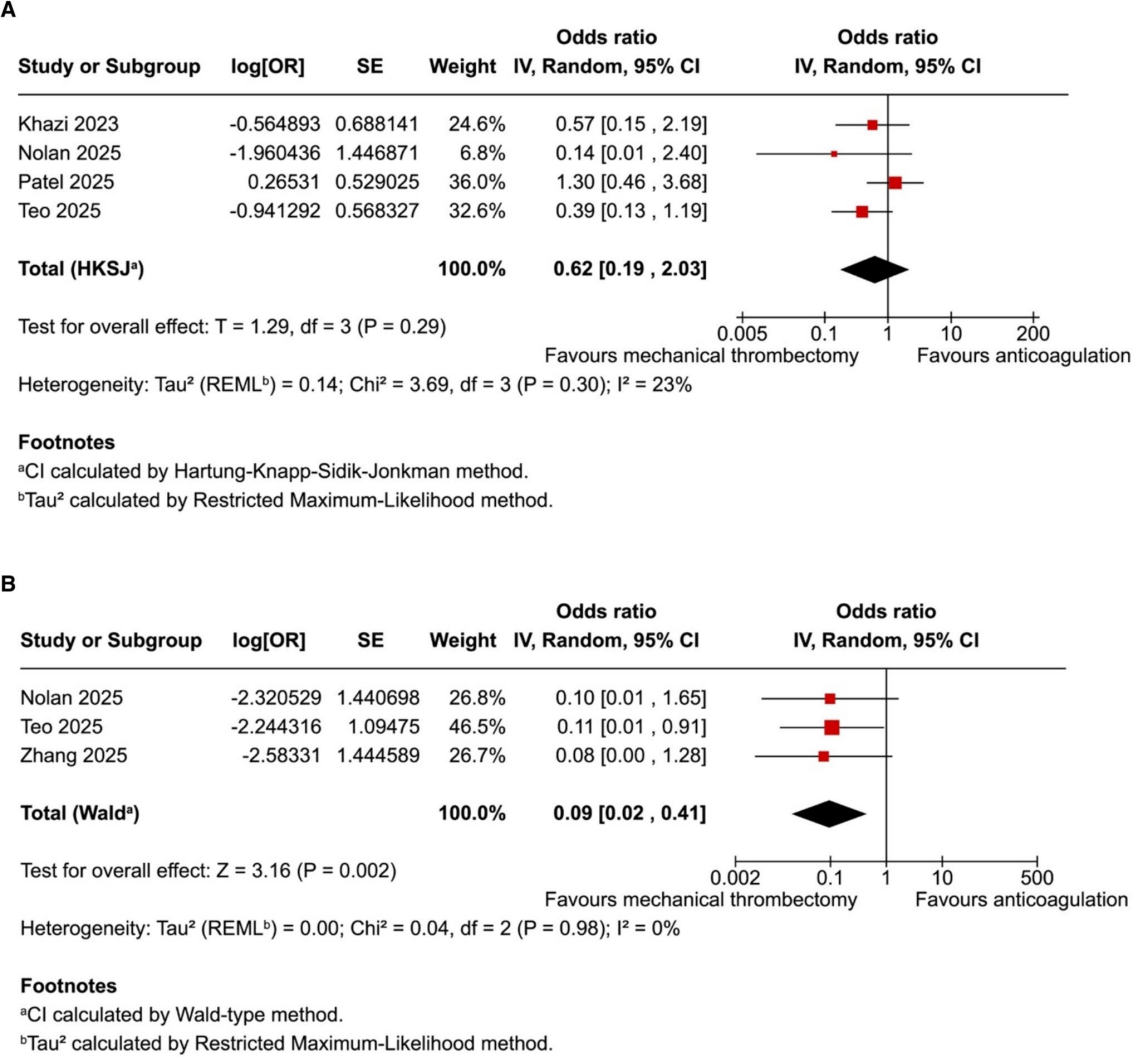
**A**: In patients with intermediate-risk pulmonary embolism, the incidence of all-cause in-hospital mortality was not significantly different between mechanical thrombectomy and anticoagulation. **B**: The incidence of all-cause 30-day mortality was significantly lower with mechanical thrombectomy compared to anticoagulation

**Fig. 3:**
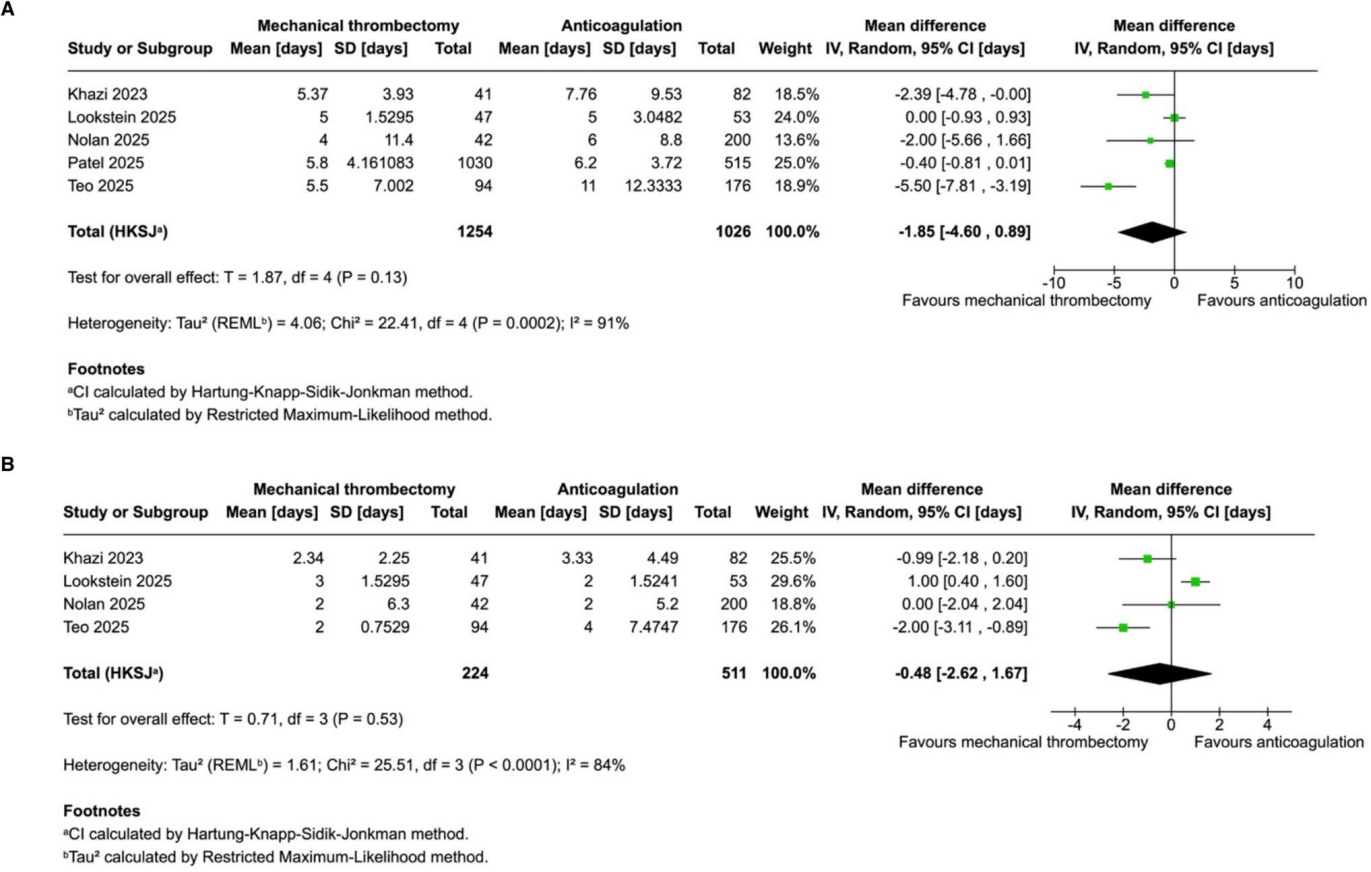
**A**: In patients with intermediate-risk pulmonary embolism, hospital length of stay was not significantly different with mechanical thrombectomy compared to anticoagulation. **B**: ICU length of stay was not significantly different with mechanical thrombectomy compared to anticoagulation

In patients receiving mechanical thrombectomy, there was an overall trend towards decreased all-cause in-hospital mortality (OR 0.62; 95% CI 0.19–2.03; *p* = 0.29; *I*^2^ = 23%; Fig. 2A) and significantly lower all-cause 30-day mortality (OR 0.09; 95% CI 0.02–0.41; *p* = 0.002; *I*^2^ = 0%; Fig. 2B). These two mortality outcomes both showed low heterogeneity between studies, with *I*^2^ < 25%. One study [9] was excluded from the mortality analyses as it reported no deaths in either treatment group, and hence, it was not possible to calculate an odds ratio. There was no significant difference between mechanical thrombectomy and anticoagulation in terms of hospital length of stay (mean difference − 1.85 days; 95% CI − 4.60 to 0.89 days; *p* = 0.13; *I*^2^ = 91%; Fig. 3A) and ICU length of stay (mean difference − 0.48 days; 95% CI − 2.62 to 1.67 days; *p* = 0.53; *I*^*2*^ = 84%; Fig. 3B). Importantly, there was high heterogeneity in hospital length of stay and ICU length of stay between studies, with *I*^2^ > 75% in each.

### Sensitivity Analysis

Given the low heterogeneity observed for all-cause in-hospital mortality (*I*^2^ = 23%) and all-cause 30-day mortality (*I*^2^ = 0%), leave-one-out sensitivity analyses were not performed for these outcomes. For hospital length of stay and ICU length of stay, given heterogeneity was high, leave-one-out sensitivity analyses were performed. For hospital length of stay, the direction of pooled effect favoured mechanical thrombectomy across analyses. When each study was omitted in turn, in three cases the result remained non-significant, and in two cases the result was a statistically significant reduction in hospital length of stay with thrombectomy. For ICU length of stay, leave-one-out sensitivity analyses demonstrated inconsistent results, with three cases favouring mechanical thrombectomy and one case favouring anticoagulation. This variability suggests the overall pooled estimate was sensitive to the inclusion of individual studies, indicating uncertain robustness. The full results for leave-one-out sensitivity analyses for hospital length of stay and ICU length of stay are shown in Supplementary Material, section 2.

### Quality Assessment

The single randomised controlled trial [8] was assessed as low overall risk of bias. All observational studies [9, 11–13, 18, 26] were assessed as having moderate risk overall. Among the observational studies, four [9, 11, 18, 26] were assessed as having low risk of confounding and two [12, 13] were assessed as having moderate risk of confounding. Analysis of each risk of bias domain for all studies is shown in Supplementary Material, section 3.

Publication bias was assessed using funnel plot analysis. For all-cause in-hospital mortality and all-cause 30-day mortality, funnel plots showed a symmetric distribution of studies which converged towards the pooled treatment effect as weights increased, suggested no evidence of publication bias. For hospital length of stay and ICU length of stay, funnel plots suggested a possible tendency for lower-weight studies to favour mechanical thrombectomy over anticoagulation. While these findings could indicate publication bias, other causes, such as random variation and differences in baseline characteristics, should be considered. The results of these analyses should be taken with caution, as the interpretation of these funnel plots was severely constrained by the small number of included studies. Funnel plots for each outcome are shown in Supplementary Material, section 4.

## Discussion

In this systematic review and meta-analysis of seven studies and 2699 patients, we compared mechanical thrombectomy to anticoagulation in patients with intermediate-risk PE. The main findings with mechanical thrombectomy include: (1) a lower observed incidence of all-cause 30-day mortality, though this result is largely driven by observational data and should be interpreted with caution; (2) a non-significant trend towards lower incidence of all-cause in-hospital mortality with low heterogeneity between studies; (3) a modest trend towards lower hospital length of stay that was not statistically significant but remained consistent with sensitivity analysis; and (4) no significant difference in ICU length of stay, which was characterised by high heterogeneity between individual studies.

The therapeutic landscape for intermediate-risk PE is rapidly evolving, with several emerging treatments being described in recent studies [5, 27]. Systemic thrombolysis is associated with a modest benefit in a composite endpoint of mortality and haemodynamic compensation, but a significant increase in rates of intracranial and major extracranial bleeding [4]. Consequently, there has been growing interest in alternative perfusion strategies which may be associated with fewer adverse events. One such approach is reduced-dose thrombolysis, which seeks to maintain reperfusion efficacy while minimising the risk of major haemorrhage associated with full-dose regimens [28, 29]. Ongoing trials are underway and will provide important insights into this [29]. Catheter-based therapies are another important group of emerging therapies [5]. Catheter-directed thrombolysis may reverse right ventricular dysfunction more effectively compared to anticoagulation alone in intermediate-risk PE based on several randomised studies [27, 30, 31]. However, these trials have primarily analysed surrogate endpoints as opposed to patient-centred clinical endpoints [5]. Mechanical thrombectomy has more recently emerged as an alternative endovascular approach. In contrast to catheter-directed thrombolysis, mechanical thrombectomy does not involve the administration of thrombolytic medications, and as such offers a theoretical advantage of reduced incidence of major bleeding. The PEERLESS trial specifically compared mechanical thrombectomy with catheter-directed thrombolysis in patients with intermediate-risk PE [7]. In this study, mechanical thrombectomy demonstrated statistical superiority over catheter-directed thrombolysis based on a hierarchical win ratio analysis, with a win ratio of 5.01 (95% CI 3.68–6.97; *P* < 0.001). However, this advantage was driven primarily by lower rates of clinical deterioration and/or escalation to bailout therapy (1.8% versus 5.4%; *P* = 0.04) and lower rates of postprocedural ICU admission (41.6% versus 98.6%; *P* < 0.001). Importantly, there were no statistically significant differences in all-cause mortality (0.0% versus 0.4%; *P* = 1.00), intracranial haemorrhage (0.7% versus 0.4%; *P* = 0.62) or major bleeding (6.9% versus 6.9%; *P* = 1.00) between treatment groups. Therefore, while the PEERLESS trial suggests mechanical thrombectomy may confer certain clinical and operational advantages over catheter-directed thrombolysis, it does not necessarily confer a mortality or safety benefit in this population. Furthermore, interpretation of the existing literature is complicated by substantial heterogeneity in the devices used, limiting comparability across studies. Prior meta-analyses have examined mechanical thrombectomy or catheter-based therapies more broadly in PE populations. For example, Husseiny et al. [17] reported lower all-cause mortality with mechanical thrombectomy compared with anticoagulation; however, their analysis included high-risk PE patients, in whom systemic thrombolysis remains standard of care [1]. Similarly, Zoumpourlis et al. [15] evaluated catheter-based therapies collectively and demonstrated reductions in short- and mid-term mortality. However, these analyses combined heterogeneous interventions, including catheter-directed thrombolysis and mechanical thrombectomy, which differ mechanistically and may have distinct clinical outcomes [7]. In contrast, our meta-analysis specifically isolates mechanical thrombectomy and restricts inclusion to intermediate-risk PE, a group in whom the optimal reperfusion strategy remains uncertain. In our meta-analysis, mechanical thrombectomy was associated with a possible reduction in 30-day mortality compared to anticoagulation.

Major adverse events, including rates of major bleeding, were not reported consistently enough in our meta-analysis to permit pooled estimates. Nonetheless, evidence from individual studies suggests that postoperative complications following mechanical thrombectomy remain relatively low [7, 8]. Reported rates of major bleeding ranged from 1.1 to 6.9% [7, 8, 11], broadly comparable to those observed with anticoagulation [8, 11]. Similarly, rates of clinical deterioration requiring rescue therapy with mechanical thrombectomy have been generally low in existing studies, ranging from 0 to 2.1% [7, 8, 12], slightly lower than those reported with anticoagulation [8, 12], although no single study has demonstrated a statistically significant difference. Long-term outcomes, including the development of chronic thromboembolic pulmonary hypertension (CTEPH) and patient quality of life, have not been systematically evaluated, limiting assessment of the enduring safety profile of mechanical thrombectomy. Further data are needed to better characterise adverse events and to weigh these risks against the procedural benefits when considering mechanical thrombectomy in PE.

Current clinical guidelines on treatment of intermediate-risk PE provide cautious recommendations regarding the use of catheter-based procedures, largely due to the limited evidence base [1, 3]. However, it is important to note most guidelines predate recent large registries and randomised trials in this area [8, 11, 18]. The 2019 ESC/ERS guidelines [3] offer a weak recommendation to consider catheter-directed treatments in patients with intermediate-risk PE who experience haemodynamic deterioration despite anticoagulation, while explicitly highlighting the small sizes of existing randomised trials and scarce data on clinical efficacy endpoints. The 2020 American Society of Hematology guidelines suggest catheter-directed thrombolysis in selected patients with extensive deep vein thrombosis (DVT), but make no recommendations regarding mechanical thrombectomy in PE, reflecting ongoing uncertainty [3]. Overall, current guidelines remain circumspect regarding mechanical thrombectomy in intermediate-risk PE. The findings of the present study contribute to the growing body of literature in this area and underscore the need for further randomised trials in this area.

Our findings have several important clinical implications. Although largely based on observational data, our results suggest that mechanical thrombectomy may be associated with improved short-term outcomes compared with anticoagulation in patients with intermediate-risk PE. Notably, several included studies [8, 18, 26] focused on patients with intermediate–high-risk PE, possibly reflecting a clinical preference to intervene in individuals at higher risk of deterioration. While this is certainly plausible, we could not conduct a subgroup analysis on intermediate–high-risk PE exclusively due to the limited number of available studies. Numerous factors influence the accessibility of the use of mechanical thrombectomy [5]. The adoption of multidisciplinary pulmonary embolism response teams (PERT) has greatly increased the use of catheter-directed reperfusion strategies in PE management [5, 32]. Nevertheless, important practical barriers remain, including uncertainty regarding cost-effectiveness and the variation in available thrombectomy devices which require clinical training and institutional support [5].

Our study has important limitations. Firstly, six of seven studies were non-randomised. Although all the observational studies included employed methods to account for confounding—including multivariate analysis, propensity score matching and inverse probability treatment weighting—and were assessed as having low to moderate risk of confounding, the inherent limitations of observational data remain. In clinical practice, patients selected for mechanical thrombectomy may differ systematically from those receiving anticoagulation in ways not fully captured by these adjustments, including unmeasured clinical severity, comorbidities or institutional treatment preferences. In addition, the small number of randomised studies meant it was not possible to perform subgroup analyses on randomised data alone. Secondly, hospital length of stay and ICU length of stay were associated with very high heterogeneity (*I*^2^ = 91% and *I*^2^ = 84%, respectively), substantially limiting the interpretability of the pooled estimates. Leave-one-out sensitivity analyses suggested directional consistency for hospital length of stay; however, this was not the case for ICU length of stay. These findings are likely influenced by institution-specific factors including local discharge practices, ICU admission thresholds, peri-procedural care pathways and resource availability. As such, length-of-stay outcomes should be interpreted cautiously and may not be generalisable across healthcare settings. Finally, assessment of right ventricular dysfunction could not be pooled across individual studies given variability in definitions, outcome measures and timing of assessment. Individual studies reported metrics such as RV/LV ratio, tricuspid annular plane systolic excursion (TAPSE), right ventricular outflow tract volume time integral (RVOT VTI), as well as invasive measurements such as mean and systolic pulmonary artery pressure. However, no single right heart outcome was reported consistently enough to permit a pooled assessment.

## Conclusion

In summary, the results of this meta-analysis including over 2,500 patients with intermediate-risk PE show that mechanical thrombectomy may be associated with a lower incidence of all-cause 30-day mortality compared to anticoagulation alone. However, this finding is driven predominantly by observational data and small event numbers and should therefore be interpreted cautiously. All-cause in-hospital mortality, hospital length of stay and ICU length of stay did not differ significantly between groups. Overall, these results are hypothesis generating and highlight the need for adequately powered randomised controlled trials to better define the role of mechanical thrombectomy in intermediate-risk PE.

## Supplementary Information

Below is the link to the electronic supplementary material.

Supplementary file1 (DOCX 2457 kb)

## Abbreviations

CTEPH: Chronic thromboembolic pulmonary hypertension
DVT: Deep venous thrombosis
ESC: European Society of Cardiology
ERS: European Respiratory Society
PE: Pulmonary embolism
PERT: Pulmonary embolism response team
PRISMA: Preferred reporting items for systematic reviews and meta-analyses
RVOT VTI: Right ventricular outflow tract velocity time integral
TAPSE: Tricuspid annular plane systolic excursion
