# Supplementary material for: Mechanical Thrombectomy Versus Anticoagulation in Intermediate-Risk Pulmonary Embolism: A Systematic Review and Meta-Analysis
Source: Cardiovasc Intervent Radiol. 2026 Mar 22;49(6):1094–104. doi: 10.1007/s00270-026-04423-5 (PMC13212738; doi:10.1007/s00270-026-04423-5)

Supplementary material

# 1. Search strategy

## 1.1. PubMed

((pulmonary embolism OR pulmonary embolus) AND (intermediate risk OR submassive OR sub-massive)) AND (thrombectomy OR embolectomy) AND (anticoag* OR coagulation factor inhibit* OR thrombin inhibit* OR antithromb* OR clotting inhibit* OR warfarin OR Coumadin OR vitamin K antagonist OR heparin OR enoxaparin OR Lovenox OR Clexane OR low molecular weight heparin OR LMWH OR NOAC OR DOAC OR novel oral anticoag* OR direct oral anticoag* OR factor Xa inhibitor OR rivaroxaban OR Xarelto OR dabigatran OR Pradaxa OR apixaban OR Eliquis OR edoxaban OR Lixiana)

## 1.2. Embase

((pulmonary embolism OR pulmonary embolus) AND (intermediate risk OR submassive OR sub-massive)) AND (thrombectomy OR embolectomy) AND (anticoag* OR coagulation factor inhibit* OR thrombin inhibit* OR antithromb* OR clotting inhibit* OR warfarin OR Coumadin OR vitamin K antagonist OR heparin OR enoxaparin OR Lovenox OR Clexane OR low molecular weight heparin OR LMWH OR NOAC OR DOAC OR novel oral anticoag* OR direct oral anticoag* OR factor Xa inhibitor OR rivaroxaban OR Xarelto OR dabigatran OR Pradaxa OR apixaban OR Eliquis OR edoxaban OR Lixiana)

## 1.3. Cochrane

((pulmonary embolism OR pulmonary embolus) AND (intermediate risk OR submassive OR sub-massive)) AND (thrombectomy OR embolectomy) AND (anticoag* OR coagulation factor inhibit* OR thrombin inhibit* OR antithromb* OR clotting inhibit* OR warfarin OR Coumadin OR vitamin K antagonist OR heparin OR enoxaparin OR Lovenox OR Clexane OR low molecular weight heparin OR LMWH OR NOAC OR DOAC OR novel oral anticoag* OR direct oral anticoag* OR factor Xa inhibitor OR rivaroxaban OR Xarelto OR dabigatran OR Pradaxa OR apixaban OR Eliquis OR edoxaban OR Lixiana)

# 2. Leave-one-out sensitivity analyses

## 2.1 Hospital length of stay


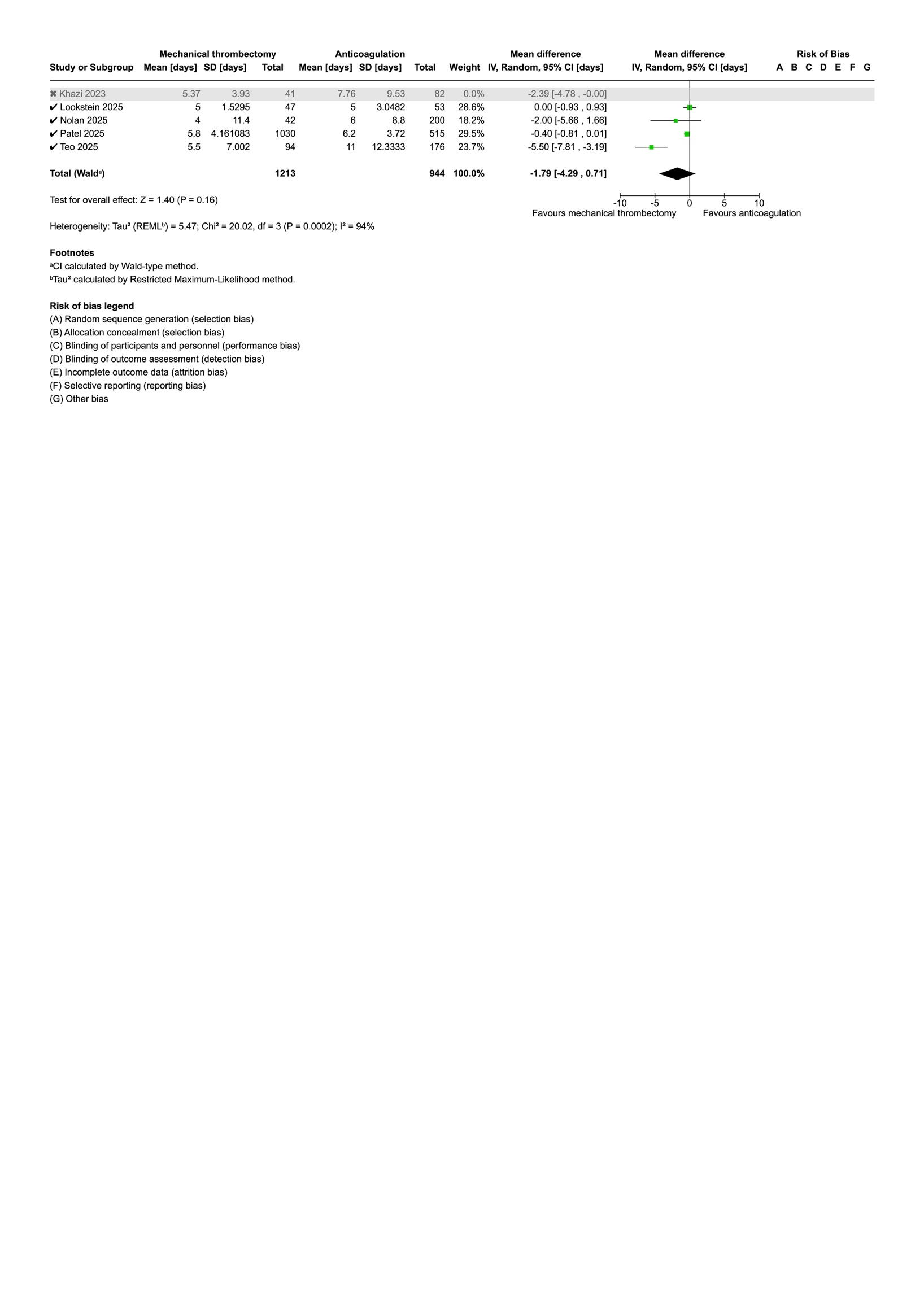

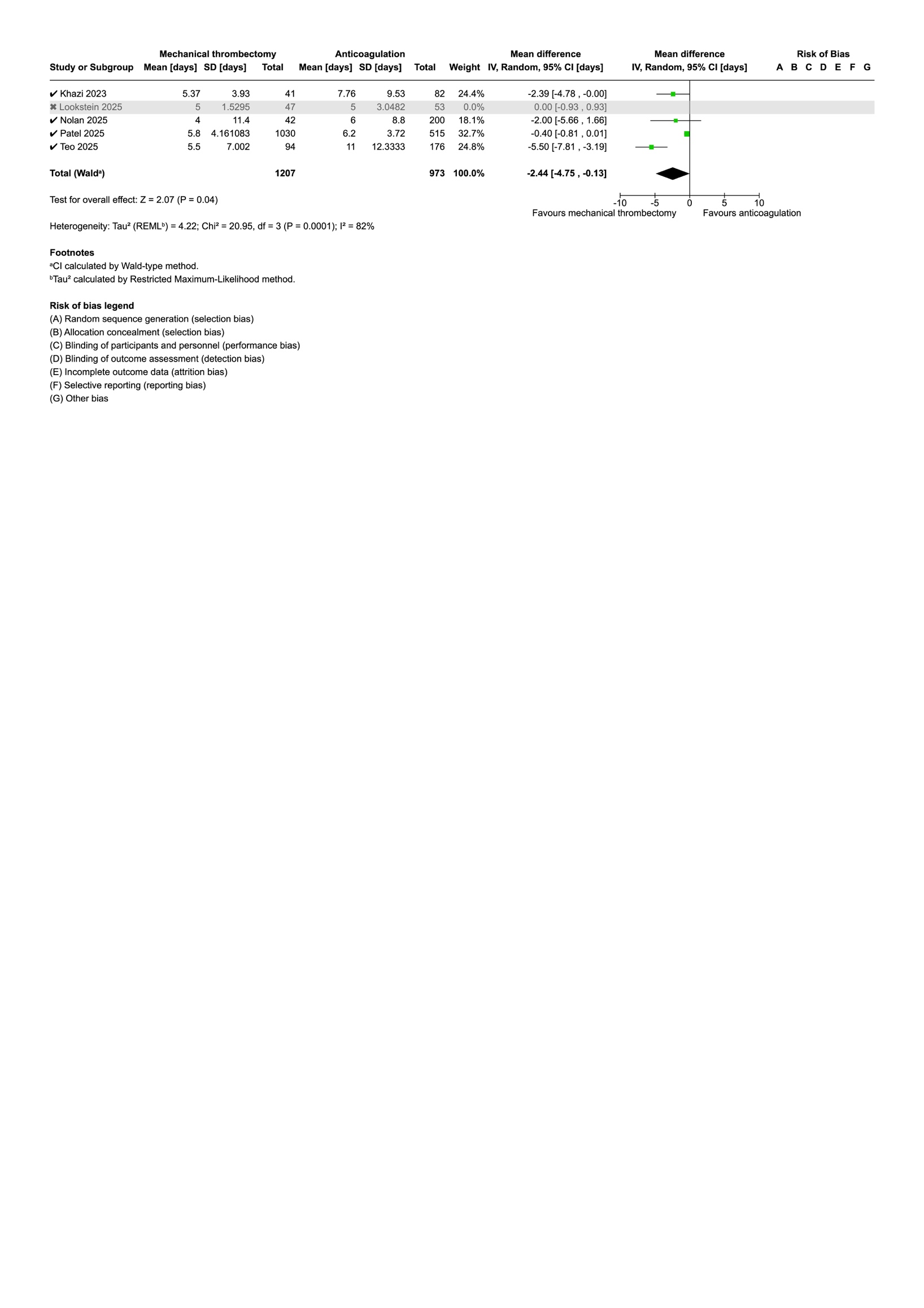

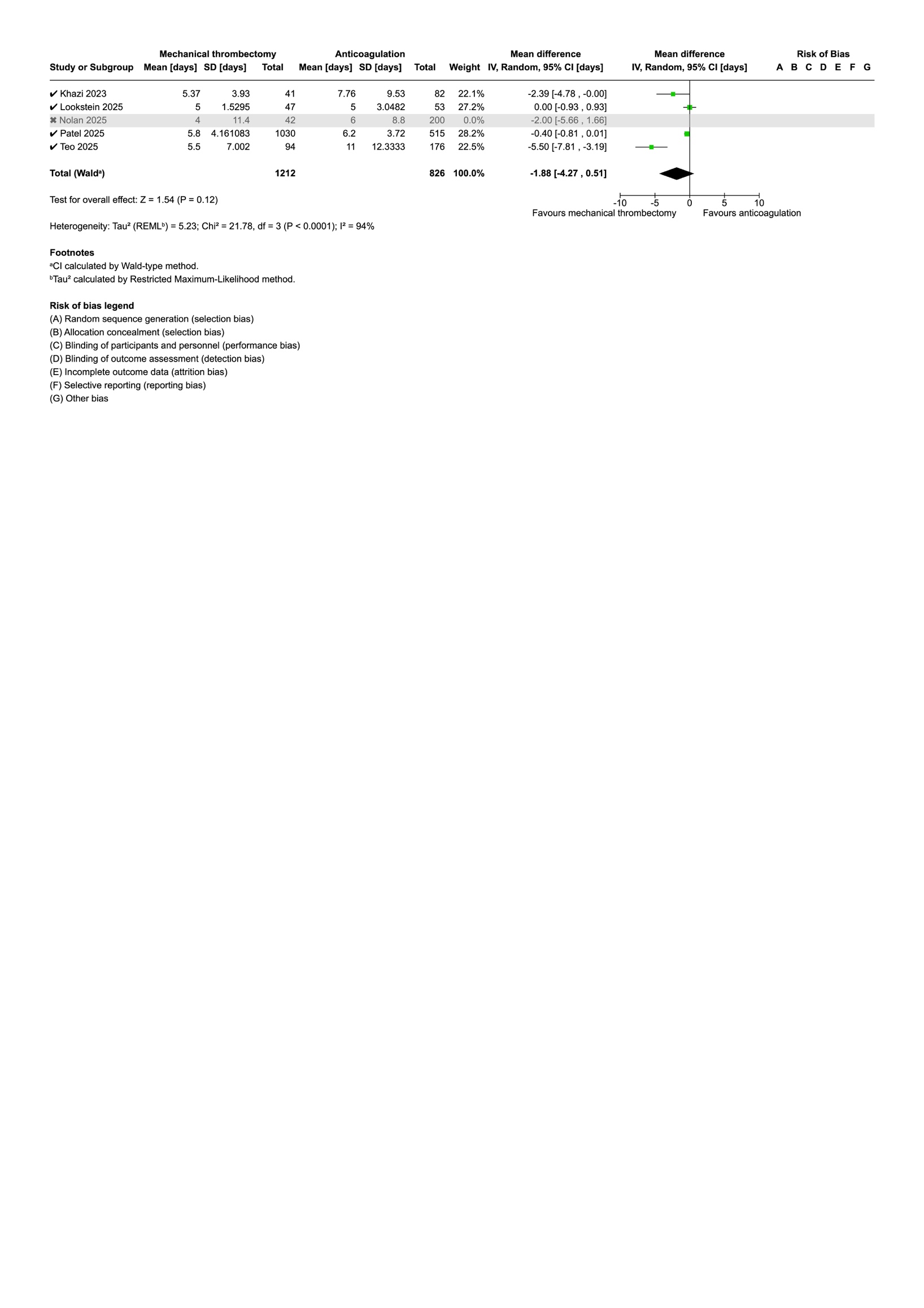

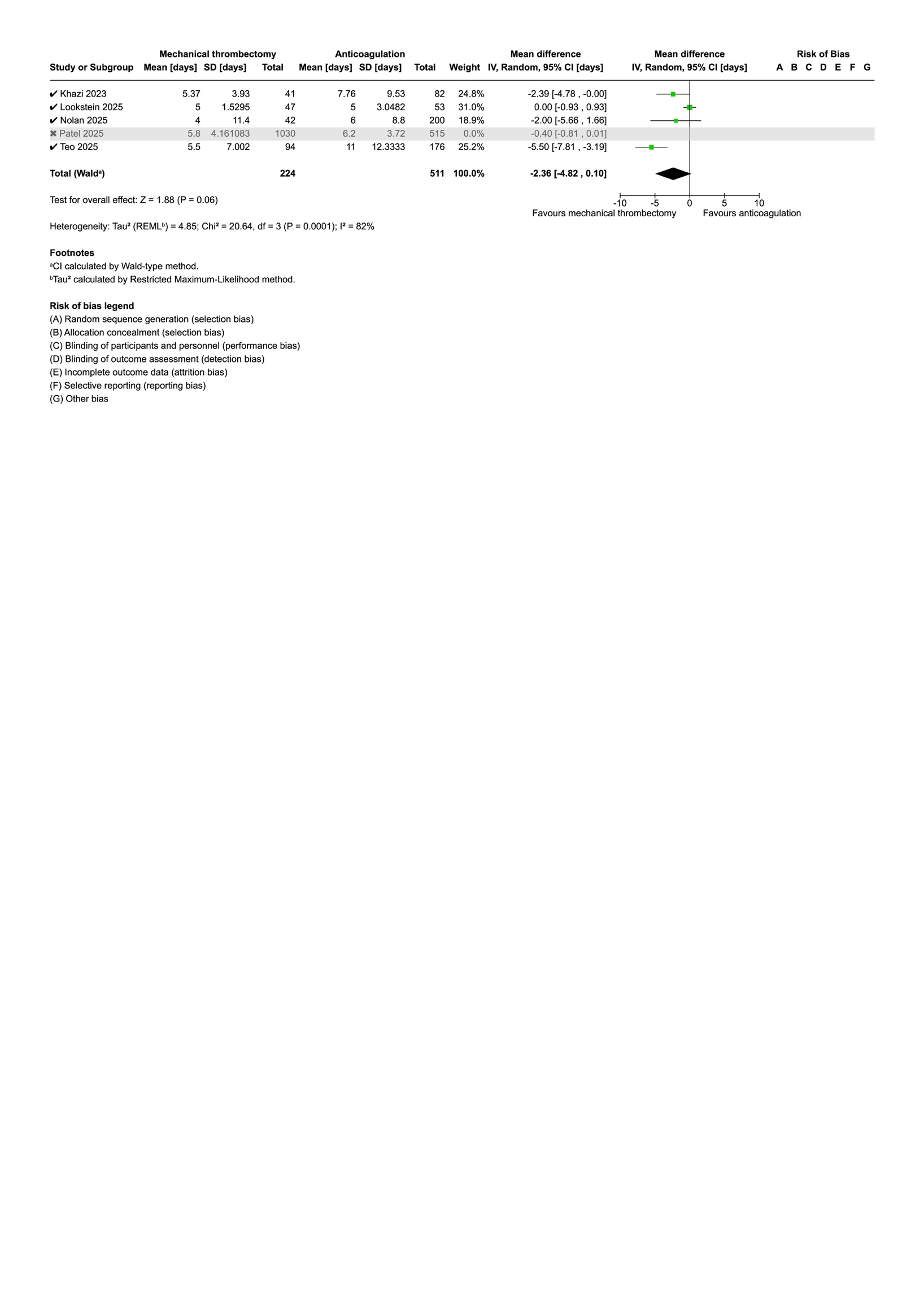

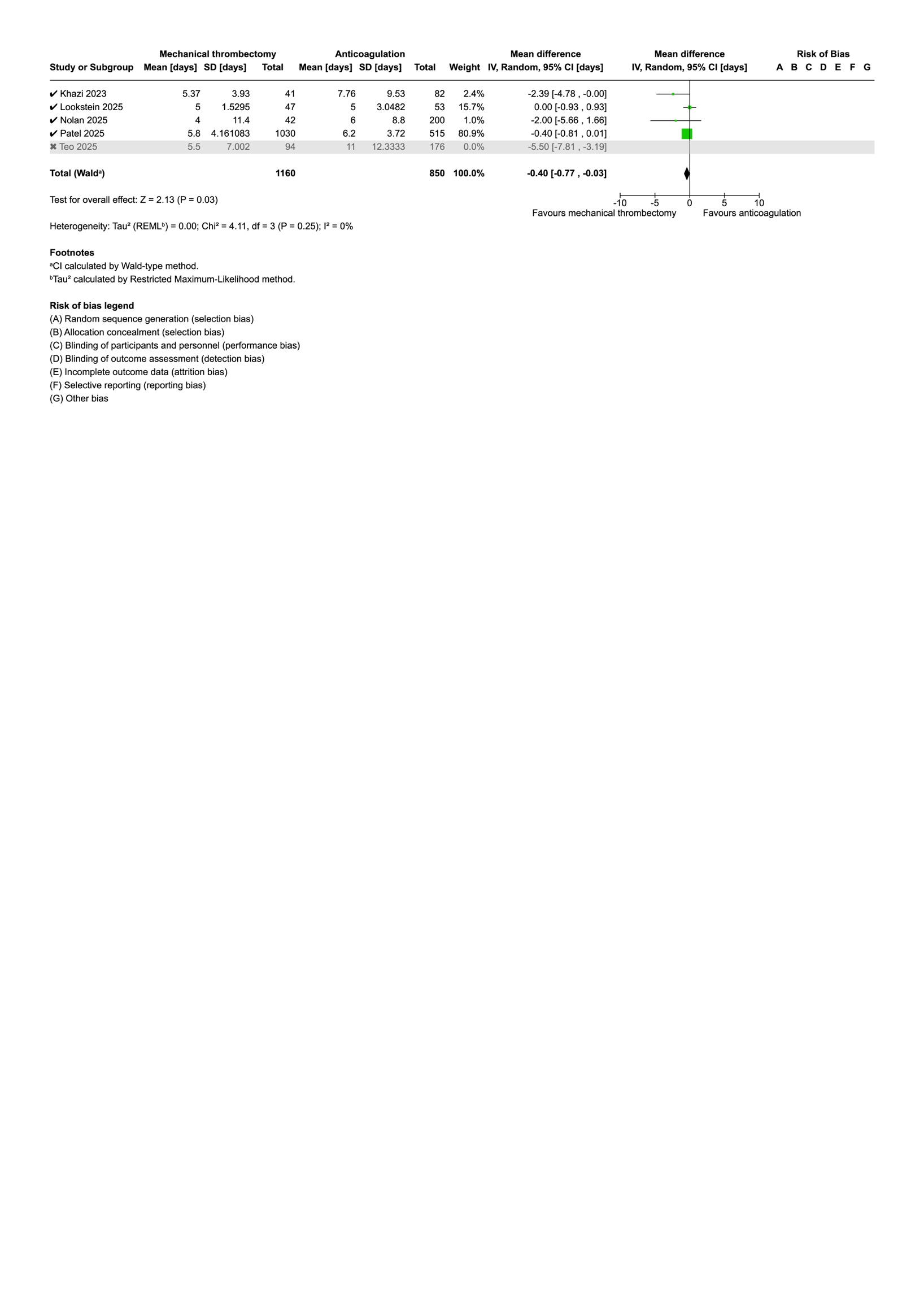


## 2.2 ICU length of stay


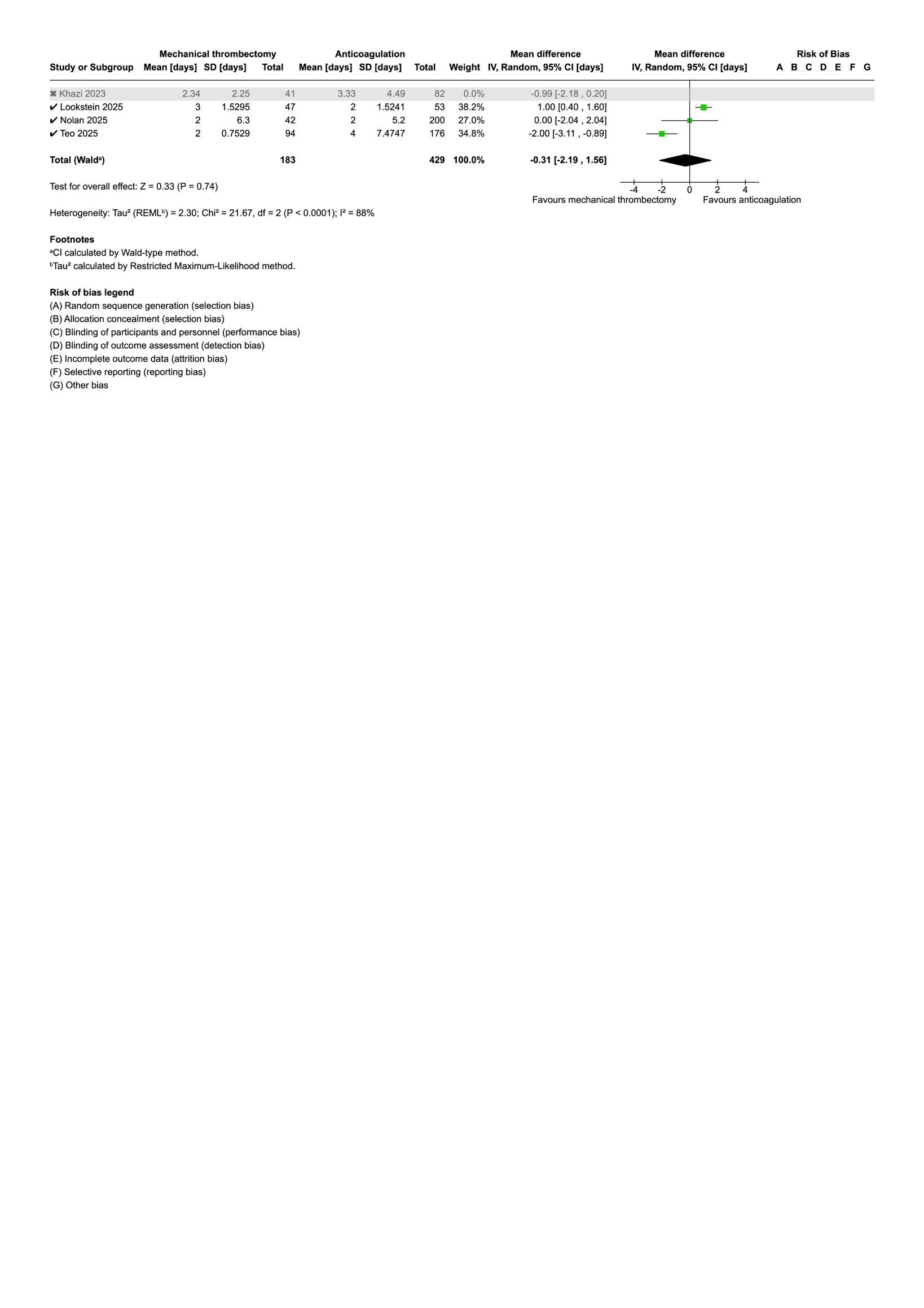

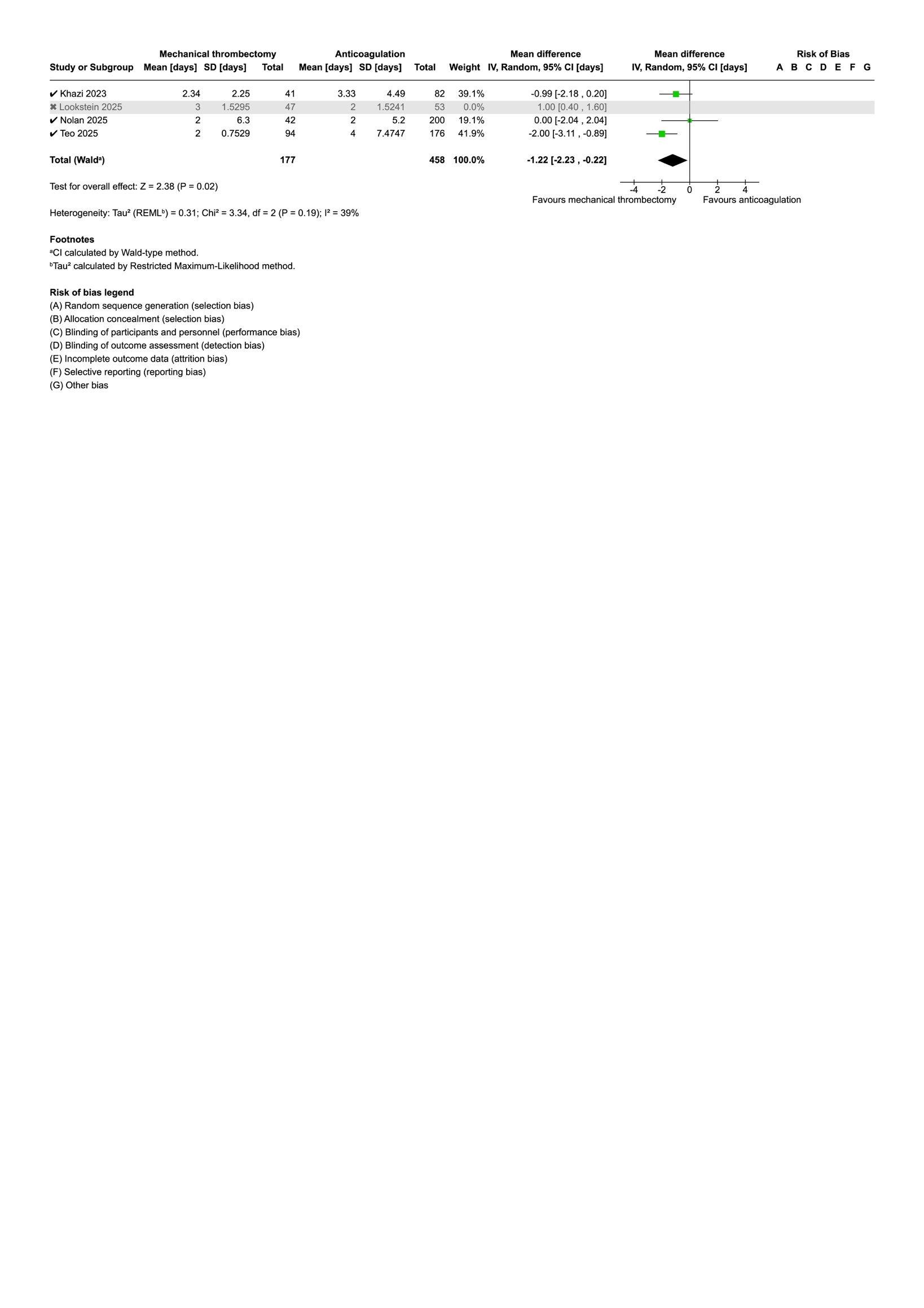

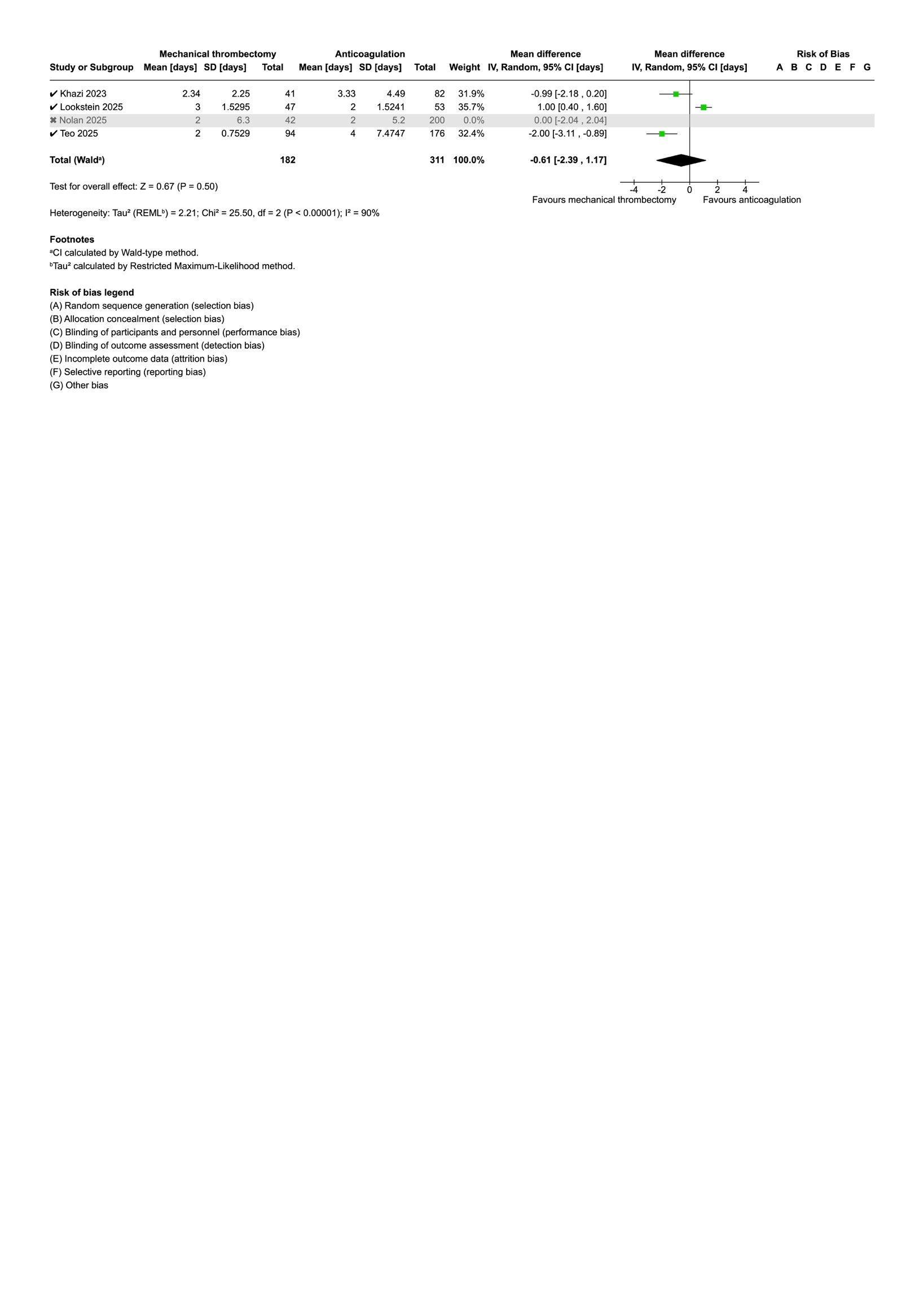

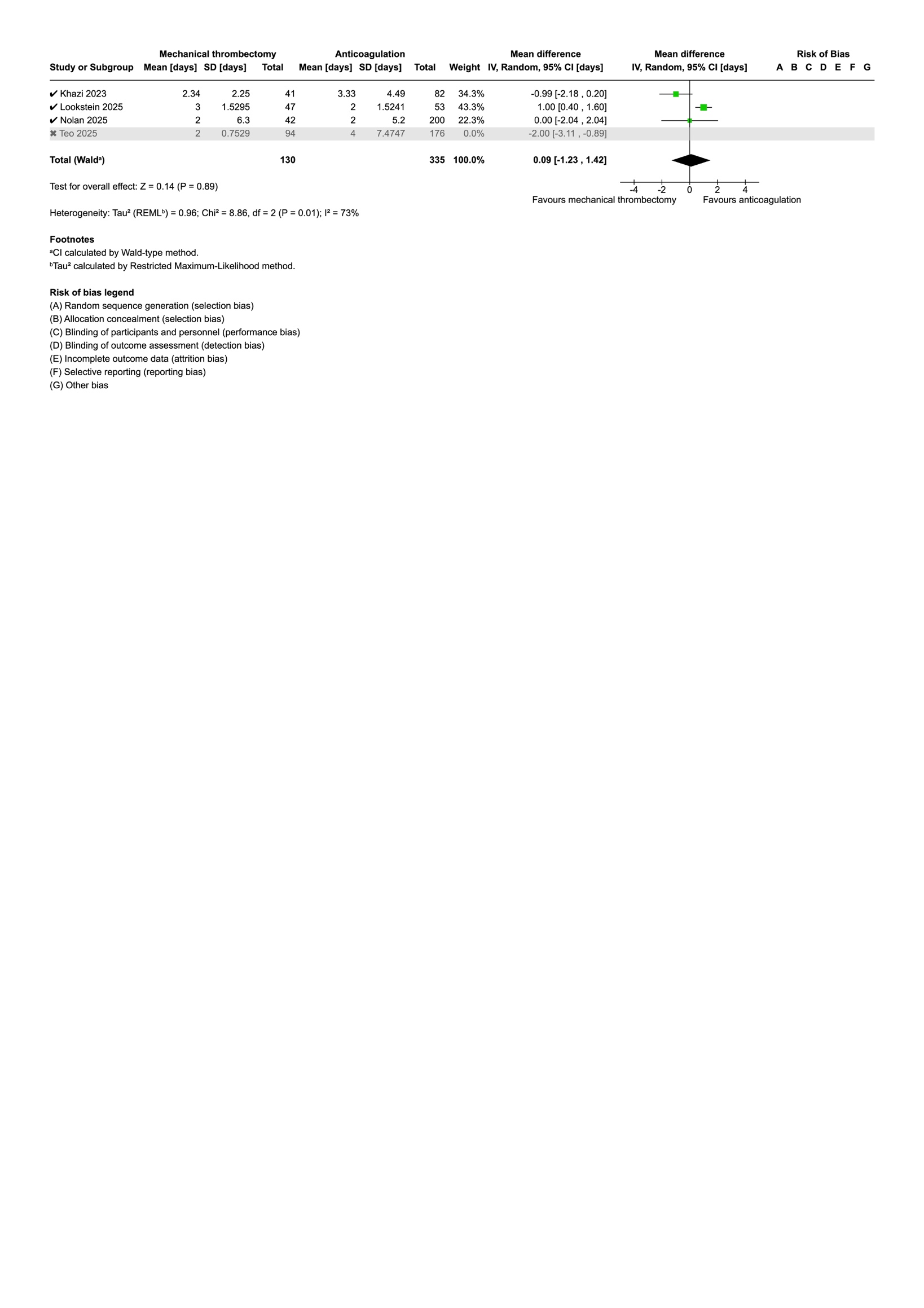


# 3. Risk of bias assessment

## 3.1. Randomised studies

Results of assessment using the RoB2 tool.

|  | **Domain 1** | **Domain 2** | **Domain 3** | **Domain 4** | **Domain 5** | **Overall** |
| --- | --- | --- | --- | --- | --- | --- |
| **Lookstein 2025** | Low risk | Low risk | Low risk | Low risk | Low risk | Low risk |

## 3.2. Observational studies

Results of assessment using the ROBINS-I version 2 tool.

|  | **Domain 1** | **Domain 2** | **Domain 3** | **Domain 4** | **Domain 5** | **Domain 6** | **Overall** |
| --- | --- | --- | --- | --- | --- | --- | --- |
| **Khazi 2023** | Low risk | Low risk | Low risk | Low risk | Moderate risk | Moderate risk | Moderate risk |
| **Nolan 2025** | Low risk | Low risk | Low risk | Low risk | Moderate risk | Moderate risk | Moderate risk |
| **Patel 2025** | Moderate risk | Low risk | Low risk | Low risk | Moderate risk | Moderate risk | Moderate risk |
| **Teo 2025** | Low risk | Low risk | Low risk | Low risk | Moderate risk | Moderate risk | Moderate risk |
| **Wang 2025** | Low risk | Low risk | Low risk | Low risk | Moderate risk | Moderate risk | Moderate risk |
| **Zhang 2025** | Moderate risk | Low risk | Moderate risk | Low risk | Moderate risk | Moderate risk | Moderate risk |

# 4. Assessment of publication bias

## 4.1. Funnel plot for all-cause in-hospital mortality


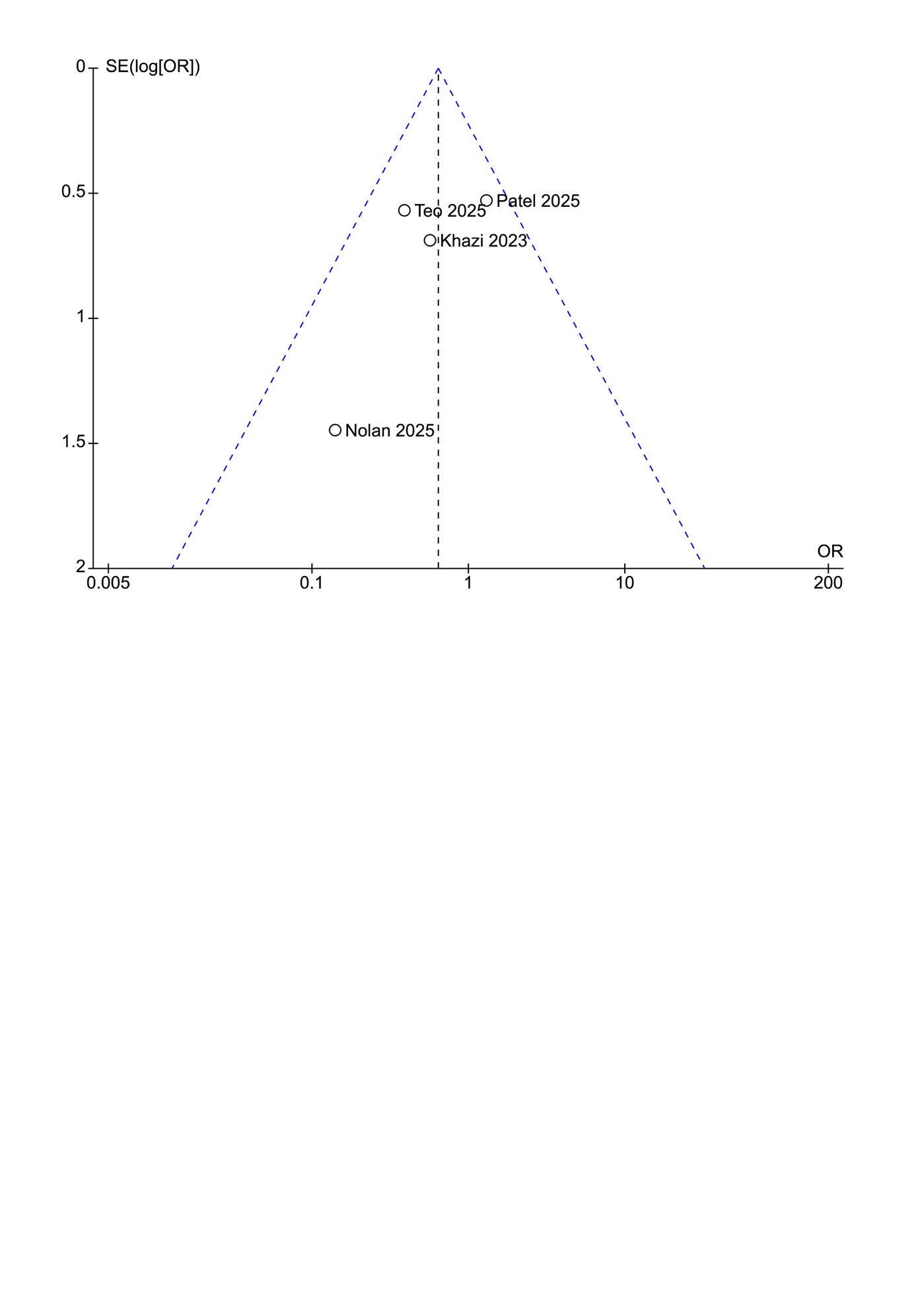


## 4.2. Funnel plot for all-cause 30-day mortality


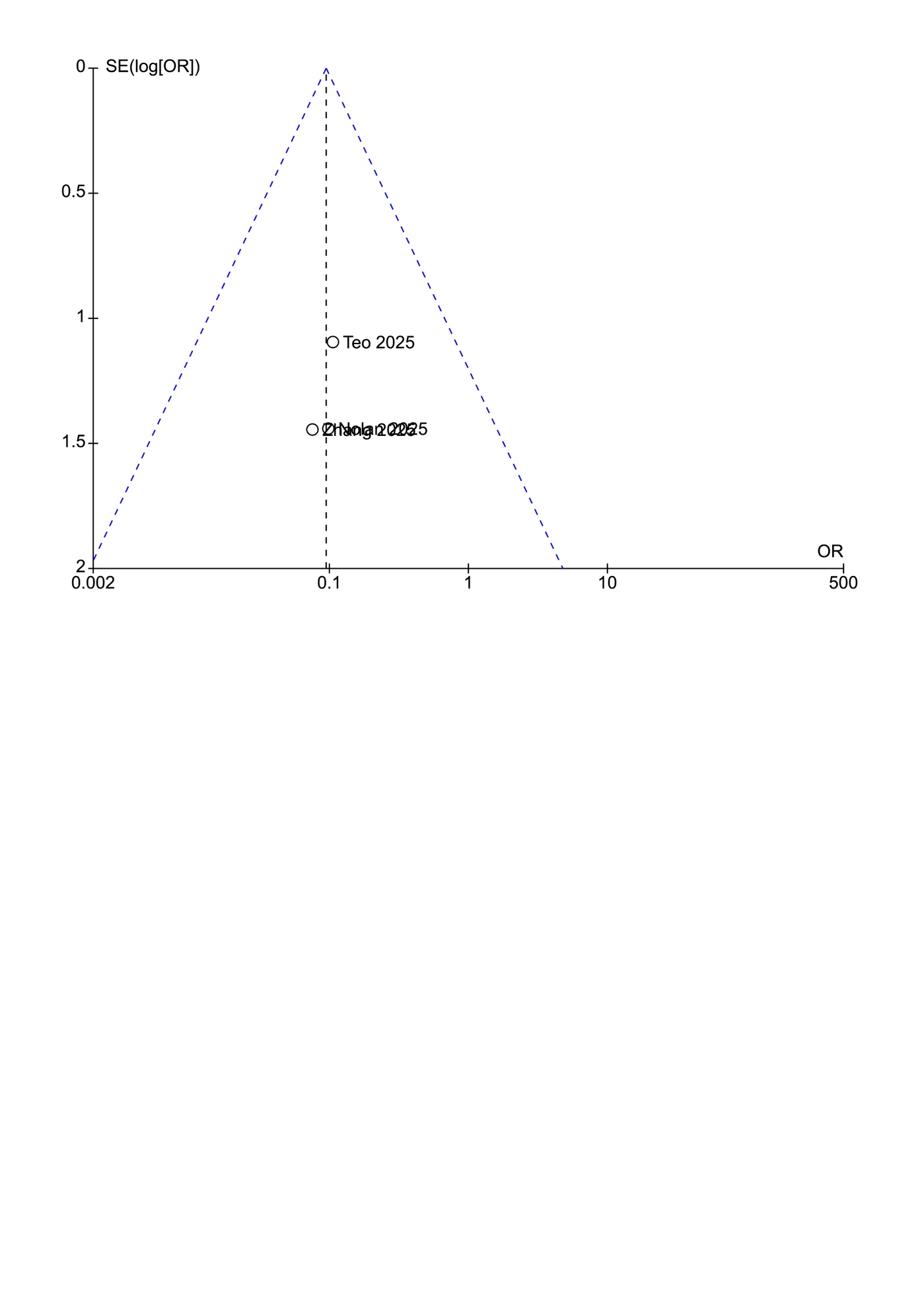


## 4.3. Funnel plot for hospital length of stay


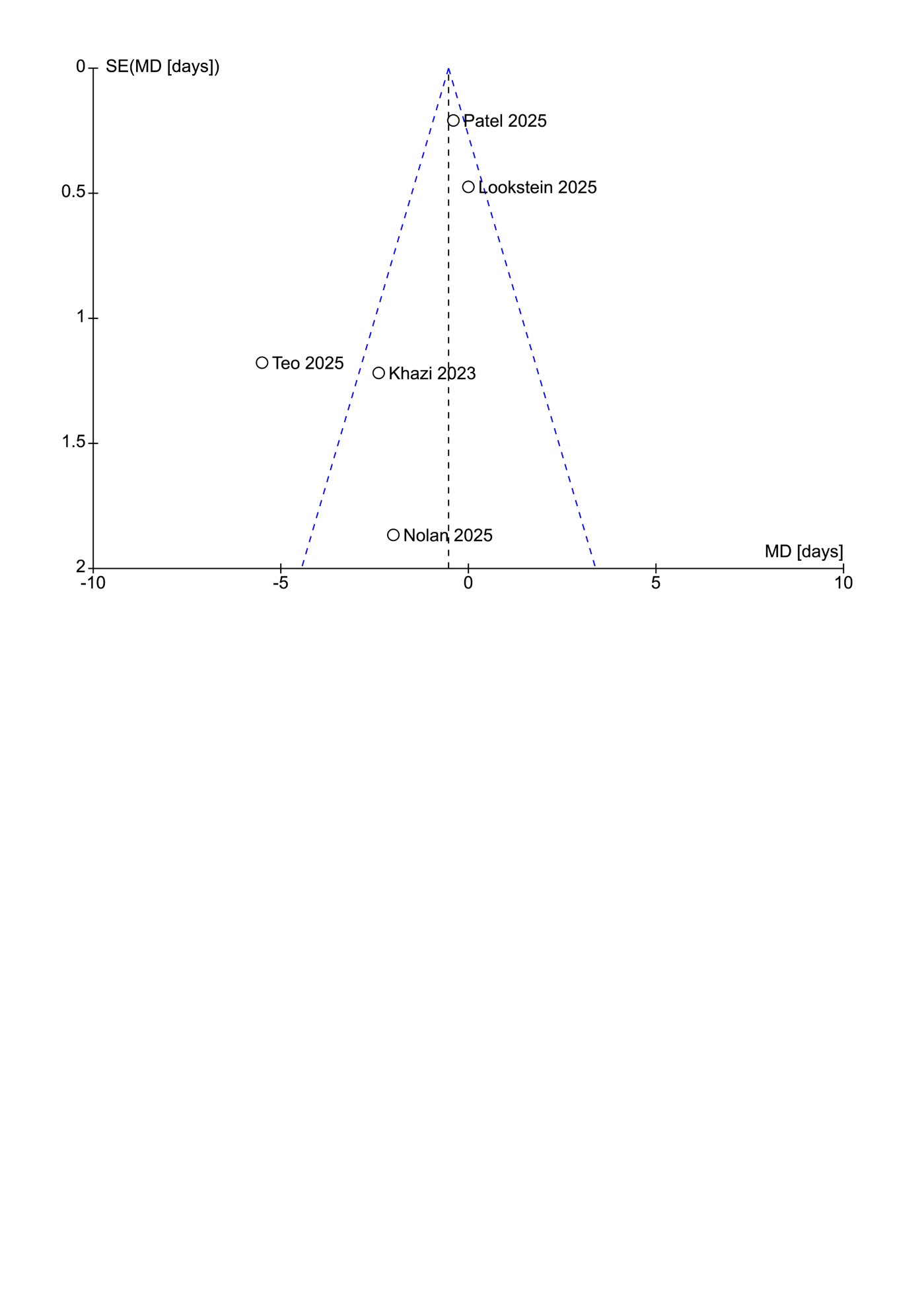


## 4.4. Funnel plot for ICU length of stay


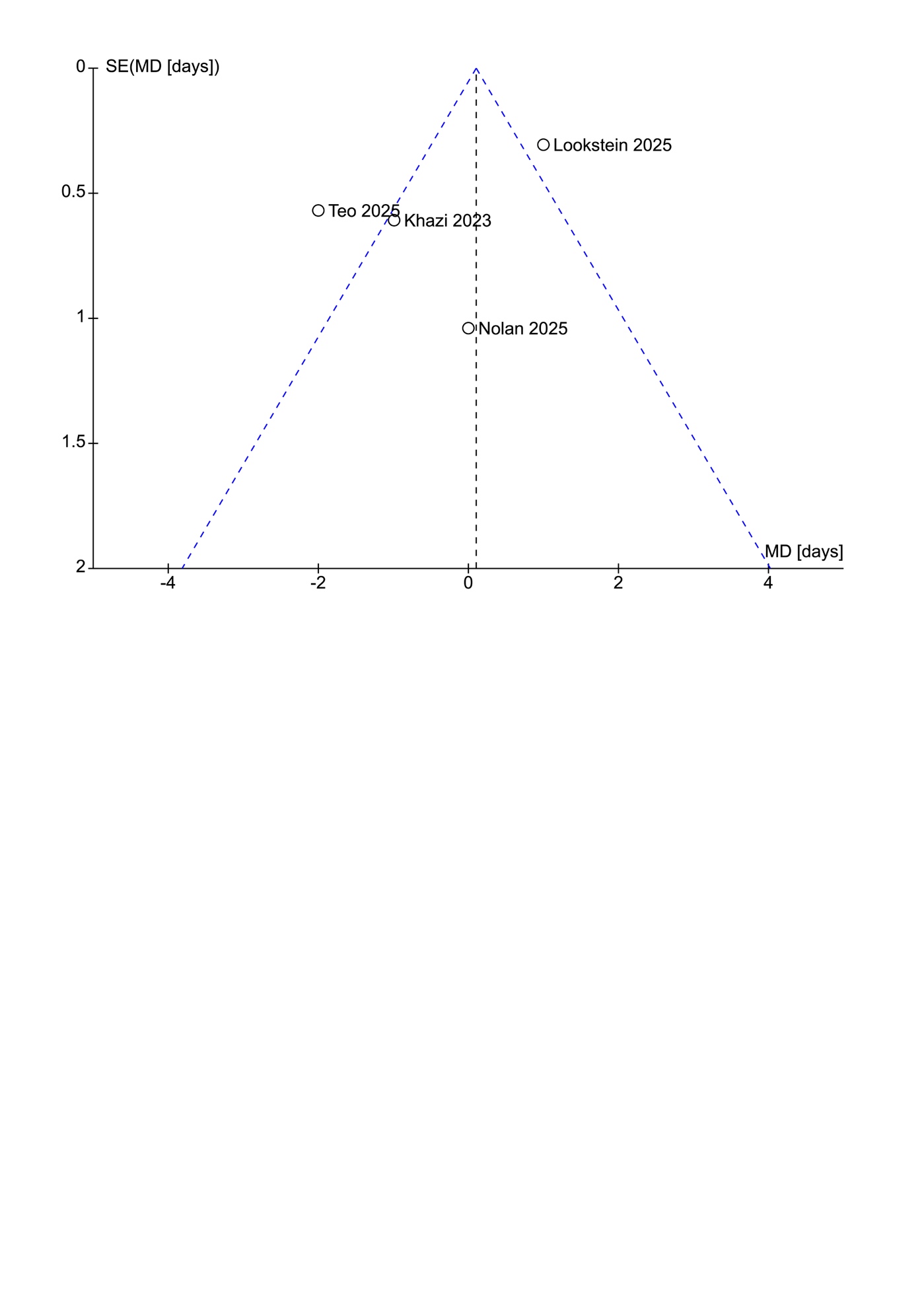

Supplement: Supplementary file 1 — Supplementary file1 (DOCX 2457 kb) [file 270_2026_4423_MOESM1_ESM.docx]
